# Identifying by Radiograph Grade 4 Aplasia of the Caudal Lamina Ventralis in the Equine Sixth Cervical Vertebra and Three Coinciding Morphological Variations

**DOI:** 10.3390/ani16030482

**Published:** 2026-02-04

**Authors:** Audrey DeClue, Kate Workman, Sharon May-Davis

**Affiliations:** 1 DeClue Equine LLC, 6955 NW 100th Street, Ocala, FL 34482, USA; 2 Rexos Incorporated (5O1c3b—Nonprofit Research Facility), 1087 Lakebay Rd., Vass, Carthage, NC 28394, USA; rexoseq@gmail.com; 3 Denali Equine PLLC, 1087 Lakebay Rd., Vass, NC 208394, USA; 4 Faculty of Science, Agriculture, Business and Law, University of New England, Armidale, NSW 2350, Australia

**Keywords:** foramen transversarium, horses, longus colli, seventh cervical vertebra, transverse process, vertebral artery

## Abstract

To date, radiographically identifying the bony landmarks relevant to diagnosing the complete C6 aplasia of the caudal lamina ventralis (C6 aCLV4) has not been described. Furthermore, a gross study has identified C6 aCLV4 as the main correlation between transposition of the CLV from C6 to C7, where coinciding neck pain was commonly reported. This study aimed to identify C6 aCLV4 in radiographs, where the outcome might benefit equine practitioners in isolating neck pain. Initially, the relevant bony landmarks were radiographically determined from a normal C6 by applying a lateral 30° dorsal–ventral oblique view. From here, the caudal border of the transverse process (TP) projecting from the vertebral body became the defining point of reference due to the image clearly demarcating the adjacent CLV. In C6 aCLV4 cases, the CLV is completely aplastic caudal to the TP. Twenty mixed-breed horses (13 males/7 females) aged 3–22 years radiographically demonstrated a C6 aCLV4, which was confirmed by their corresponding gross morphology. During this process three anomalous variations became apparent—in the longus colli muscle, C7 foramen transversarium, and vertebral artery. Therefore, this study demonstrates effective techniques for identifying C6 aCLV4 in horses, where the results might assist veterinarians in diagnosing neck pain while providing anatomical clarity.

## Simple Summary

This study aimed to identify the relevant bony landmarks in C6 radiographic images of a complete caudal absence of the lamina ventralis in affected horses. One cadaveric study identified this specific malformation as a grade 4, where the findings revealed a strong dependency in transposing the absent caudal lamina ventralis from C6 to C7’s ventral surface. In these horses, reports of neck pain were more common. Hence, the objective of this study was to radiographically determine a C6 grade 4 absent caudal lamina ventralis in a live horse only when the clinical symptoms warrant further investigation. The study revealed that the first point of reference was the connection between the caudal border of the transverse process and the vertebral body. This junction provided a guide to the cranial aspect of the caudal lamina ventralis, where its morphology could be evaluated. From here a grade could be calculated in the affected horses. The radiographic findings identified 20 cases that were confirmed by the corresponding gross morphology, during which three coinciding anomalous variations were noted—in the longus colli muscle, C7 foramen transversarium, and vertebral artery. Therefore, the results reported here might assist veterinarians in the diagnosis of neck pain and/or indicate the need for further diagnostic imaging.

## 1. Introduction

In equids, the sixth cervical vertebra (C6) is atypical due to its unique lamina ventralis, a bony ridge that extends along the lateroventral border and is regarded as one of three branches of the transverse process [1,2,3,4]. This osseous structure, as with many others, has been subjected to an extensive, subjective nomenclature over the last 150 years as equine anatomists grappled with the evolving and inconsistent terminology [4,5]. When considering the term lamina ventralis, there are historical discrepancies, for example, the third or ventral process of the transverse process, transverse process tritubercular, and tricuspid process [6,7,8]. In 1910, Sisson’s [4] illustration of the ventral cervical vertebrae noted the lamina ventralis on C6 and credited the German anatomist Reinhold Schmaltz with the work. By 1924, Schmaltz [9] had labeled the lamina ventralis as separate from the cranial and dorsal transverse process branches, referring to it as ‘crista ventralis lateralis’, which anatomically translates to ‘ridge in the bone’. This deviation in terminology might have derived from paleologists discussing that the crista ventralis lateralis was in fact a rudimentary fused rib forming a specialized modification in C6 [10,11]. Even so, veterinary textbooks to this day still present variable terminology, such as ventral tubercle, ventral process, and lamina ventralis [4,12,13]. So, for the purpose of this study, the term lamina ventralis will be referenced as per *Nomina Anatomica Veterinaria* [14].

In Sisson [4], it states “the 3rd branch of the transverse process and fossa was sometimes absent or reduced on one side”; thereafter, this quote was replicated in associative veterinary textbooks [4,5,15]. Now, questions surrounding the aplasia of the C6 caudal lamina ventralis (C6 aCLV) are widely debated in the scientific community [16,17,18,19,20,21,22] and on social media. For veterinarians, the use of social media by pet owners as a source of information on many subjects is concerning, especially when the source is not verifiable [23,24,25,26,27,28]. When the first reports of C6 aCLV appeared in the 1800s, they were recorded as incidental findings [29,30]; now, in the modern era with improved radiographic technology and protocols [19,31], diagnosed cases nominating breeds have been reported in significant numbers [16,17,18,19,22,32,33]. Furthermore, owners of diagnosed horses are describing the experiences, symptoms, behaviors, and triggers on social media. This is where veterinarians can take an active role in establishing transparent mechanisms of diagnosis by addressing owners’ concerns with accurate, science-based diagnostic evidence and guidance [28,34].

In C6 aCLV cases, there is a disparity among authors regarding the clinical relevance, which, in effect, has generated a conflict of opinions. Most regard the aCLV and coinciding malformations as relevant [16,17,18,19,20], while others have reported no clinical relevance [21,22]. Even so, neck pain appears to be the most common associative finding among those authors in the affirmative [16,17,18,19,20], with Henderson et al. [20] observing the transposition of the aCLV from C6 to C7 as the primary correlation with pain. These outcomes have led to a consensus among authors that further research is necessary, especially as the complexity of the gross morphology relevant to the cervicothoracic malformations are predominantly contingent on a C6 aCLV [19,35,36,37,38,39]. Hence, relating the potential clinical relevance to each morphological presentation requires accurate diagnostic protocols. This involves further research that specifically targets the morphological features that coincide with congenital malformations. For example, Ros et al. [19] reported a positive association among the C6 aCLV, transposition to C7, and congenital malformations of the first and second sternal ribs, while May-Davis et al. [38] reported a strong dependency between the size of the C6 aCLV and the transposition to the ventral surface of C7.

Yet, even with a plethora of scientific publications on the subject, only two have specifically addressed the diagnostic radiographic protocols for congenital malformations of the cervicothoracic junction [19,31]. The authors of these studies reported that the C6 aCLV presented as either a unilateral or bilateral aplasia, with concerns that a bilateral presentation could be misinterpreted as C5 due to the similar morphology. Gee et al. [31] confirmed the radiographic findings with the gross morphology, while Ros et al. [19] characterized the aCLV radiographs to diagrams. During this time, another study examined, through gross observation, the varying sizes of the C6 aCLV and proposed a grading system from 1 to 4, with grade 4 being complete aplastic CLV [36].

Based on these grading protocols, a subsequent study identified that a strong dependency exists between the C6 aCLV4 and the transposition of the aCLV to the ventral surface of C7 [37]. This places significant relevance on accurately diagnosing the grading of a C6 aCLV4, especially with its association with transposing to C7 and its coinciding anomalous morphology. Even though replication of the foramen transversarium in C7 and displacement of the longus colli have been previously reported [4,5,15], recent studies have linked these anomalies to the C6 aCLV4, along with deviations in the scalene muscles and tracheal deformation along the dorsal ridge [19,36,37,39]. However, except for Gee et al. [16], the findings from most studies are based on either radiographs or gross morphology, with no radiographic correlation. This leaves a gap in the research, where the key linking factor in transposition to C7 is a C6 aCLV4 and yet, no comparative radiographic images have confirmed or correlated this grade with the corresponding gross morphology.

Therefore, as radiographic protocols are well established for most equine conditions [31], the objective of this study is to build on previous studies and establish the precise bony landmarks for the diagnostic examination of a C6 aCLV4 malformation. This outcome will be achieved through comparative radiographic imagery to gross morphology. In addition, to eliminate repetitive anatomical studies through dissections and to maximize the learning opportunity, two previously reported coinciding morphological variations will be simultaneously investigated in the presence of these osseous variations. These are the longus colli muscle [38,40], the anomalous foramen transversarium in C7 [5,6,15,30,37], and the vertebral artery due to its relationship with the foramen transversarium in cervical vertebrae. Ultimately, the aim is to aid veterinarians in the accurate reporting of a C6 aCLV4 in live animals via radiographs, while assessing the potential influence of associative structures, and by extending the diagnostic examination to C7 if the aplastic C6 CLV is indicative of transposition. In effect, the findings might benefit the correlation of radiographic information to possible clinical relevance, especially considering reports of coinciding neck pain and therefore in turn, provide a clearer prognosis for the equine patient.

## 2. Materials and Methods

### 2.1. Ethical Statement

No horses were euthanized for the purpose of this study. The reasons for euthanasia were compiled from the patient medical records and are referred to in Table 1. Each horse was donated by the owner after careful veterinary examination and discussion, and in each case, the horse was examined by 2 or more veterinarians premortem, and 1 or more of the authors postmortem. In all cases of euthanasia, the American Veterinary Medical Guidelines for Euthanasia of Animals was followed.

The study spanned two years, from 2023 to 2025.

### 2.2. Terminology

The terms lamina ventralis, foramen transversarium, and longus colli muscle are derived from *Nomina Anatomica Veterinaria* (2017) [14], while transverse process and vertebral artery are derived from Getty [15].

### 2.3. Normal and Anomalous Anatomy

The descriptions of normal and anomalous anatomy are derived from Getty [15], Sisson [41], Bradley [30], and Rombach [42].

#### 2.3.1. Caudal Lamina Ventralis

The caudal lamina ventralis is referred to as the 3rd branch of the transverse process, and it is a thick, almost sagittal plate that forms with its fellow of the opposite side. This branch is known to sometimes be absent or reduced on one side [15].

#### 2.3.2. Longus Colli Muscle

A two-part bundle muscle that covers the ventral surfaces of the vertebrae from the 5th or 6th thoracic vertebrae to the atlas (C1).

Origin: The thoracic part—the vertebral bodies from T1 to T5 or T6. The cervical part—transverse processes of the cervical vertebrae.
Insertion: The thoracic part—the bodies and transverse processes of the last 2 cervical vertebrae. The cervical part—the bodies of the cervical vertebrae and the ventral tubercle of the atlas [41].

The bundles of the longus colli muscle are further described by Rombach et al. [42]. The cervical part comprises of short (medial layer spans 2 intervertebral joints from the midline to the TP), long (medial layer spans 3 intersegmental joints from the midline to the TP, C2 to C5), superficial (superficial layer spans 3 intersegmental joints from the midline to the TP, C2 to C5), deep (midline bundle spans 4 intersegmental joints, C2 to C6), and short costovertebral (caudal C6—spans 2 intersegmental joints filling the ventral concavity of C7, C6 to T1). The thoracal part comprises of a long tendon with parallel fibers spanning from C6 and C7 to T5/6.

However, Bradley [41] mentions that a bundle might pass over one or more intersegmental joints than previously described.

#### 2.3.3. Anomalous C7 Foramen Transversarium

**Figure 1:**
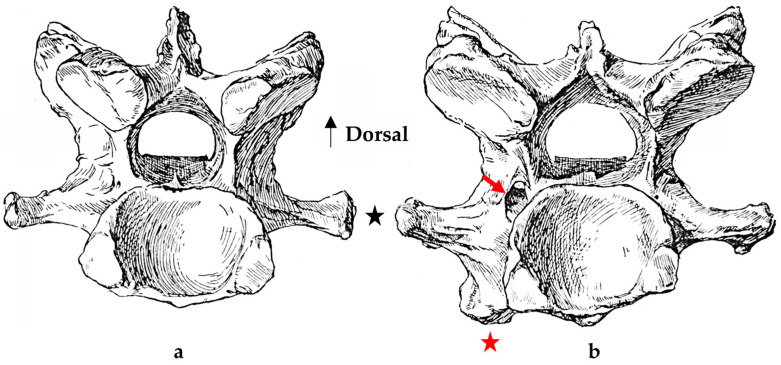
The caudal view of C7. (**a**) Normal morphology—transverse process (black asterisk). (**b**) Left-side lateroventral projection indicative of transposition from a C6 aplastic caudal lamina ventralis (red asterisk) and replication of the foramen transversarium (red arrow). Adapted from Gorton [30].

In normal C7 anatomy, foramen transversarium are not present. However, there are historical accounts of a foramen transversarium being present on one side, but rarely on both [5,6,15,30,37] (Figure 1).

### 2.4. Materials

To be eligible for this study, only those horses with suspect C6 aCLV4 radiographs were selected and allocated a prefix—‘R’ for Rexos Incorporated USA and ‘A’ for Australia.

To ensure the capture of clear radiographic images, horses with ‘R’ and ‘A’ prefixes were sedated with detomidine hydrochloride (Dormosedan^®^, Zoetis US, Kalamazoo, MI, USA), while in addition the ‘R’ horses received butorphanol tartrate (Torbugesic^®^, Zoetis US, Kalamazoo, MI, USA) for the procedure.

The radiographic equipment for the horses identified with an ‘R’ prefix utilized a portable Sound Imaging Next II, MinRay 90 KVP generator (5-10-5 Koishikawa, Bunkyo-ku, Tokyo, Japan). The horses identified with an ‘A’ prefix utilized a Radincon Porta 100 HF High Frequency portable x-ray unit, with a kV range of 40–100 kV, an mA range of 20–30 mA, and an mAs range of 0.3—20 mAs (Sydney, NSW, Australia).

Two methods of euthanasia were utilized that followed the American Veterinary Medical Association guidelines—AVMA Guidelines for the Euthanasia of Animals (2020). Intrathecally, 2% lidocaine hydrochloride (20 mg/mL) (New York, NY, USA) was administered at 2.6–4 mg/kg into the subarachnoid space located midline in the atlanto-occipital joint. Or a gunshot (caliber—22 long or magnum) to the cranium was administered in such a way as to enter the brain. The point of entry should be at the intersection of 2 imaginary lines, each drawn from the outside corner of the eye to the center of the base of the opposite ear.

### 2.5. Methods

**Figure 2:**
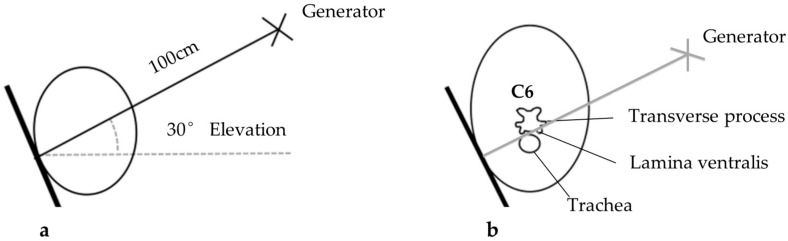
Radiographic orientation and imaging of C6 lamina ventralis. (**a**) Lateral 30° dorsal–ventral oblique view, left and right side required. (**b**) Ideal positioning for image capture and superimposition of C6 caudal lamina ventralis over trachea. Adapted from Gee et al. [16].

The sedation process was via intravenous injection into the left jugular vein. The ‘R’ and ‘A’ prefix horses received detomidine hydrochloride (Dormosedan^®^) at 0.01 mg/kg bwt., while the ‘R’ horses received additional butorphanol tartrate (Torbugesic^®^) at 0.02 mg/kg bwt. for the procedure. The radiographic orientation and depth are shown in Figure 2, where the radiographic orientation complied with a lateral 30° dorsal–ventral oblique view of C6.

**Figure 3:**
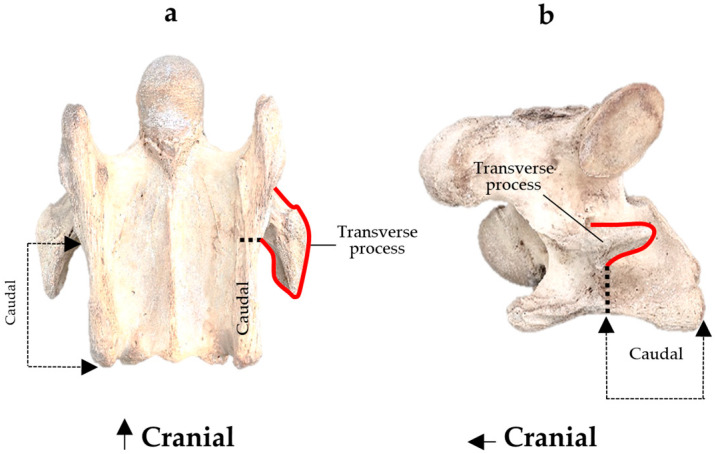
Bone view of a normal C6 anatomy. (**a**) Ventral view in a 15-year-old Oldenburg x Thoroughbred male. (**b**) Left lateral view. The red line outlines the transverse process; the black dotted line demarcates the cranial lamina ventralis from the caudal. Adapted from May-Davis et al. [21].

To aid in the process of comparing the radiographic imagery to the gross morphology, the normal ventral and lateral anatomic bone views of C6 are shown in Figure 3.

**Figure 4:**
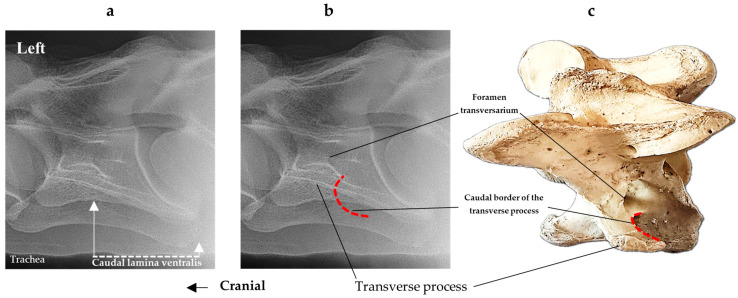
Left view of a normal C6 in a lateral 30° dorsal–ventral oblique radiographic view with the corresponding gross morphology of a Thoroughbred 14-year-old gelding. (**a**) Lateral 30° dorsal–ventral oblique radiographic view demonstrating the caudal lamina ventralis. (**b**) Radiographic view—the red dotted line denotes the caudal border of the transverse process; caudal to this border is the caudal lamina ventralis. (**c**) Gross morphological view of the bone—the red dotted line outlines the caudal border of the transverse process. The foramen transversarium is also noted.

In Figure 4, the CLV is clearly identified caudal to the transverse process in the bone view (Figure 4c). However, in the radiographic view the caudal border of the transverse process is less defined, yet the CLV is still evident (Figure 4a).

After euthanasia, removal of the soft-tissue structures associated with the caudal cervical vertebrae revealed C6 and C7, and included the detailed resection of the longus colli muscle bundles and vertebral artery, following the protocols established by Rombach et al. [26] and May-Davis et al. [27]. Removing the forelimb exposed the superficial and deep muscle layers associated with the neck for resection, including the nuchal ligament lamellae. Once the scalene muscles were removed and the perivertebral muscles were exposed, resection of the viscera (trachea, esophagus nerves, and blood vessels) along the ventral neckline exposed the longus colli muscle. This muscle is made up of intersegmental bundles that are hard to resect due to the intimate fiber arrangements and connections with the intertransversarii muscles and other longus colli bundles.

Once fully resected, C6 and C7 were disarticulated from the axial skeleton and macerated for osseous preparation, which followed the protocols established by Hangey and Dingly [29]. The bones were then placed in a cooking utensil and covered with a solution of sodium percarbonate (100 mg) and water (6 L) and warmed gently (not exceeding 80 °C) for a minimum of 3 h. By this time, the remaining structures were sufficiently softened and easily removed with a pressure hose. The bones were then rinsed in clean warm water and allowed to dry in a shady environment.

### 2.6. Radiographic Landmarks

**Figure 5:**
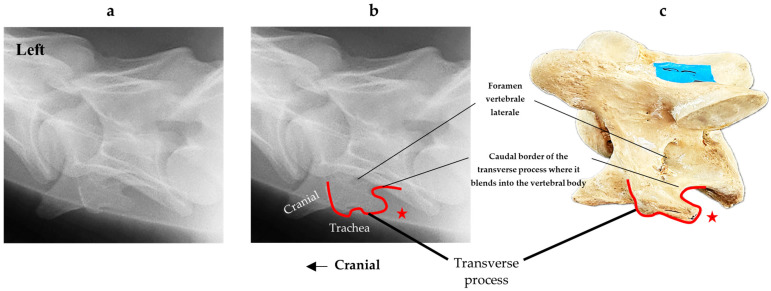
Left view of the anomalous C6 from R30 in a lateral 30° dorsal–ventral oblique radiographic view. (**a**) Radiographic view demonstrating a shortened lamina ventralis. (**b**) Radiographic view—the red line denotes the transverse process, and the red asterisk indicates the aplasia of the caudal lamina ventralis in contrast to normal. (**c**) Gross morphological view of the bone—the red line outlines the transverse process and the connection of the caudal border to the vertebral body. The red asterisk demonstrates the aplasia of the caudal lamina ventralis in contrast to normal.

Having established in a normal horse the relevant CLV bony landmarks (Figure 3 and Figure 4), the methodology thereafter was to compare the radiographs to the gross morphology. In a C6 aCLV4 horse, the radiographs must establish the caudal border of the transverse process and where it connects to the vertebral body. Caudal to this, and in contrast to a normal horse, the complete aplasia of the CLV is evident (red asterisk, Figure 5b,c). Thereafter, verification of the radiographic image was obtained by a comparison with the corresponding gross morphology to establish the efficacy of the protocols (Figure 5c).

## 3. Results

Twenty horses met the criterion and are described in Table 1 by their study prefix identification code, breed, residing country, age, gender, discipline, years ridden, height range, and reason for euthanasia.

The medical histories indicated that each horse was examined by multiple veterinarians. In each case a list of differential diagnoses (a systematic process to identify a specific disease or condition from a list of possibilities that share similar symptoms) were identified, with some horses being medically treated prior to a definitive diagnosis of caudal cervical vertebral dysfunction, which elicited further investigation.

### 3.1. Radiographic Images Comparable to Gross Morphology

By isolating the bony landmarks as described in the Materials and Methods (2.5 Radiographic Landmarks), 20 horses demonstrated via radiograph a C6 aCLV4 that was confirmed by their gross morphology (Table 2).

The C6 radiographic images and gross morphology revealed the following osseous presentations: bilateral 12/20 (R14, R15, R20, R22, R24, R26, R27, R28, R38, R39, A3, and A5); unilateral 8/20—left 6/8 (R7, R29, R30, R35, A1, and A4) and right 2/8 (R21 and A2) (Table 3). However, of the 20 confirmed C6 aCLV4 horses, 19/20 transposed the aCLV to the ventral surface of C7; A1 was the exception. The 19 transpositions corresponded in sidedness to the C6 morphology in most horses, except in R38 and A5, where the bilateral C6 transposed to the left ventral surface of C7. Size of the transposition was not consistent between the horses (Table 3).

Only 13/19 (68%) transpositions in C7 replicated the foramen transversarium (this arterial foramen is normally bilaterally present in C6), of which all except R22 and R39 (bilateral to right and bilateral to left, respectively), corresponded in sidedness to the transposition. The were 13 males compared to 7 females (Table 3).

### 3.2. Longus Colli Muscle

**Figure 6:**
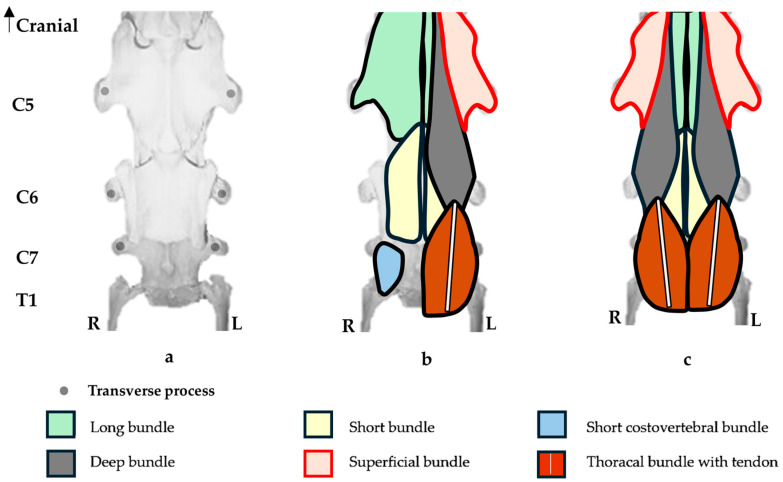
Diagrammatic ventral view of the longus colli muscle from the 5th, 6th, and 7th cervical vertebrae and partial 1st thoracic vertebra, including proximal articulation of the first sternal ribs. (**a**) Ventral view of the vertebrae. (**b**) Left view—right muscle bundles of the longus colli directly ventral to the vertebrae (1st layer). Right view—left muscle bundles of the longus colli directly overlaying the ventral layer (2nd layer). (**c**) Ventral view of the bundles of the longus colli muscle.

Normal morphology of the longus colli muscle was previously described in the Materials and Methods. Here, a brief description to recap might be applicable. The longus colli cranial attachments are from C1 to C5, and caudal to that the thoracal portion from C6. It consists of six bundles: short, long, superficial, deep, short costovertebral, and thoracal. These are diagrammatically shown in Figure 6.

**Figure 7:**
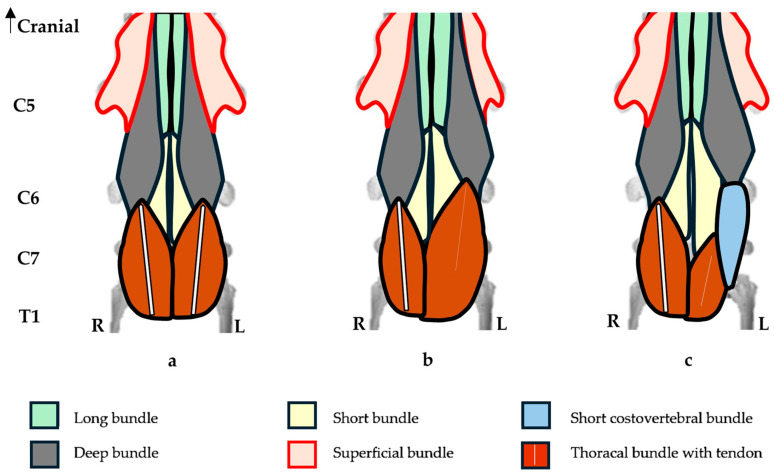
Diagrammatic ventral view of the longus colli muscle from the fifth, sixth, and seventh cervical vertebrae and partial first thoracic vertebra, including proximal articulation of the first sternal ribs. (**a**) Normal ventral view of the longus colli muscle. (**b**) Left side—unilateral C6 grade 4 aplastic caudal lamina ventralis displaying altered bundles, with a shortened deep bundle and longer hypertrophied thoracal bundle with a tendon reduced in diameter and length. (**c**) Left side—unilateral C6 grade 4 aplastic caudal lamina ventralis with transposition to the ventral surface of the 7th cervical vertebra and a transposed thoracal bundle with a tendon reduced in diameter and length. The short and short costovertebral bundles are longer.

In the one horse with a unilateral C6 aCLV4 (A1) and no transposition to C7, the morphology of the longus colli muscle was not fully examined. Instead, we utilized two bilateral cases with a single transposition to identify the unilateral C6 longus colli morphology (R38 and A5). On the affected side, the deep bundle was shortened, while the thoracal bundle was longer, hypertrophied, and its tendon displayed a reduced diameter and length (Figure 7).

**Figure 8:**
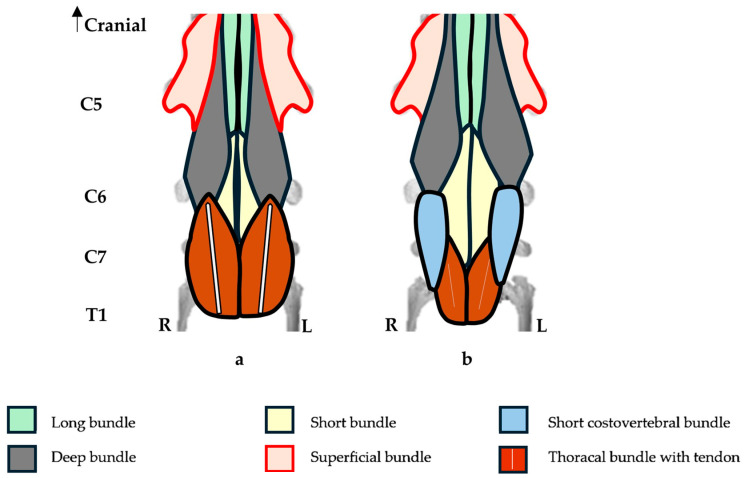
Diagrammatic ventral view of the longus colli muscle from the fifth, sixth, and seventh cervical vertebrae and partial first thoracic vertebra, including proximal articulation of the first sternal ribs. (**a**) Normal ventral view of the longus colli muscle. (**b**) Bilateral C6 grade 4 aplastic caudal lamina ventralis with bilateral transposition to the ventral surface of C7 displaying bilaterally shortened deep and thoracal bundles with tendon reduced in diameter and length. The short costovertebral bundle is bilaterally longer.

In the seven horses that demonstrated a unilateral C6 aCLV4 with corresponding C7 transposition, the longus colli bundles appeared altered. On the affected sides, and compared to the normal sides, the deep and thoracal bundles appeared shortened, with the latter attaching to the C7 transposition and the tendons displaying a reduced diameter and length. In contrast, the short and short costovertebral bundles appeared longer (Figure 8). The normal sides of C6 and C7 displayed relatively normal morphology in the attachments.

Note: In this study, the C7 vertebrae with transposition altered the thoracal and short costovertebral bundles. Here, the fibers from each bundle seemed to merge or marry with one another, making it difficult to identify the individual bundles in the resection.

In the ten horses with a bilateral C6 aCLV4 and bilateral transposition to C7, the longus colli bundles appeared altered. Here, the bilateral form replicated the unilateral arrangement on both the left and right sides (Figure 8).

### 3.3. Vertebral Artery

**Figure 9:**
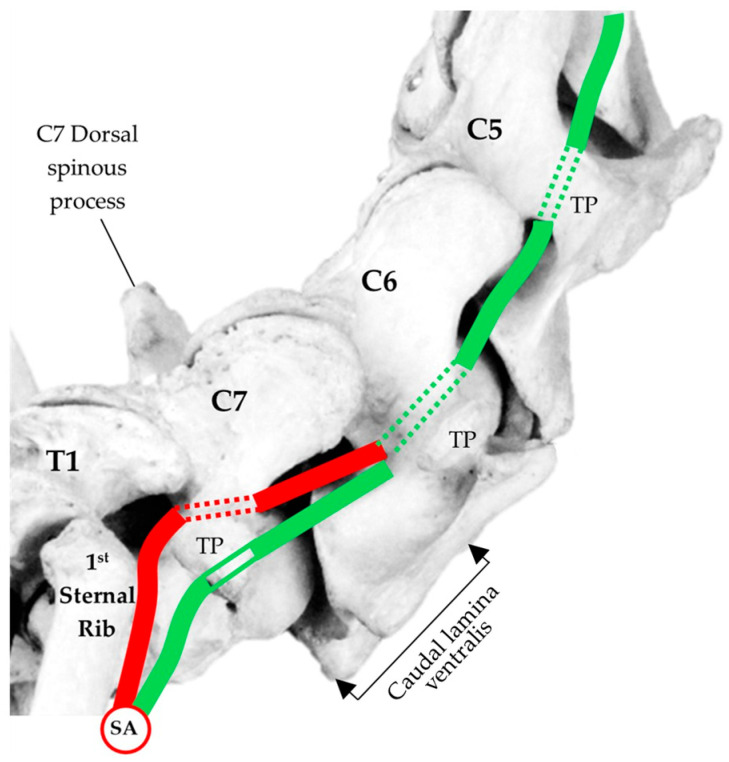
Normal right view of the vertebral artery (green) originating from the subclavian artery (SA). The two thin green lines show its passage ventral the transverse process of C7. The green dotted lines denote the normal passage of the vertebral artery through the foramen transversarium. The red line denotes the passage of the vertebral artery in those horses with a replicated foramen transversarium in the seventh cervical vertebra, with the dotted line showing the passage through the C7 replicated lateral arterial foramen. TP—transverse process.

Replication of the C7 foramen transversarium was observed in 13/19 horses (68%). This occurred in only those cases where the C6 aCLV4 was transposed to the ventral surface of C7. In these cases, the vertebral artery deviated after ascending from the subclavian artery and traversed cranial to the first sternal rib. Thereafter, the vertebral artery entered C7 via the caudal aspect of the replicated foramen transversarium, instead of the normal route to C6. This deviation was evident in the unilateral or bilateral anomalous morphology (Figure 9).

In those horses with C7 transposition and no replication of the foramen transversarium, the vertebral artery changed normal course and traversed latero-ventral to the transverse process of C7. This was due to the C7 transposition taking up the latero-ventral concavity of C7’s vertebral body, causing the vertebral artery to change course.

### 3.4. Incidental Findings

The brachial plexus demonstrated several variations from its normal passage between the dorsal and ventral scalene muscles. This included passage dorsal to the dorsal scalene, or passage through it where the muscle either longitudinally bifurcated or trifurcated. In addition, the ventral scalene often demonstrated a similar anomalous morphology, with passage of several nerves from the brachial plexus passing through these variations, for example, C8, T1, and T2.

First and second rib malformations were also observed, notably proximal and distal bifuds, which were flared and rudimentary.

**Figure 10:**
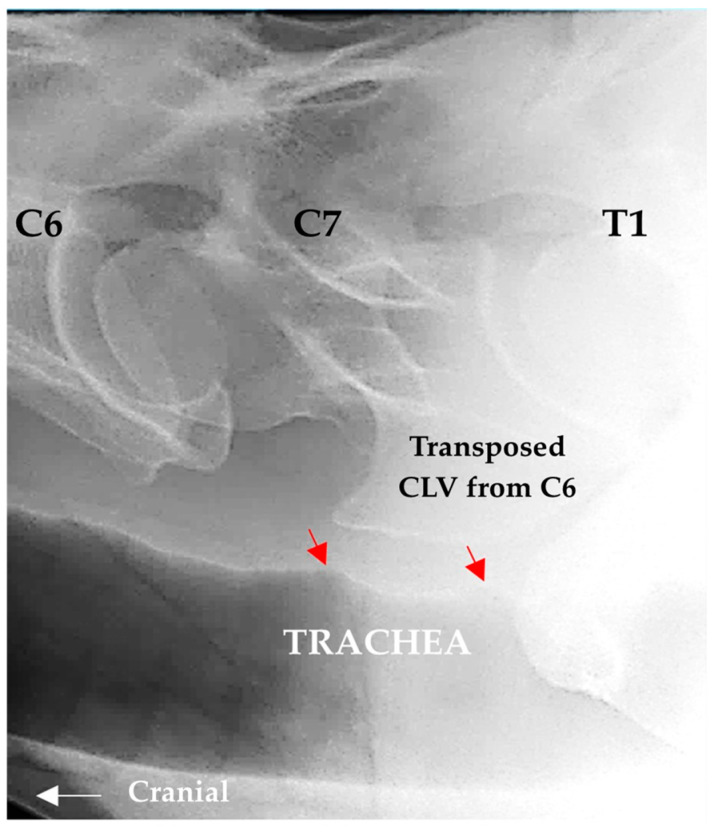
Tracheal ridge deformation ventral to the sixth and seventh cervical vertebrae in R35 (red arrows).

Although not examined in all the horses, tracheal ridge deformation was seen in the radiographic images and on the necropsy (Figure 10).

## 4. Discussion

The findings in this study support the aim of radiographically identifying a relevant bony landmark that isolates the C6 aCLV4. In addition, it provides guidelines for veterinarians when examining diagnostic images, especially as there is a strong dependency in C6 aCLV4 transposing to the ventral surface of C7 [37], where it has been reported horses are more likely to incur neck pain [16,17,20,43]. In addition, the reporting of coinciding anatomical variations in the region might assist surgeons performing procedures on or around C6/C7, such as uniportal endoscopic foraminotomy [44]. In humans, difficult or variant anatomy is a contributory factor to surgical errors [45]. Hence, anatomical clarity in the C6/C7 region might benefit equine surgeons, particularly in cases where the vertebral artery has relocated, as reported in this and a previous study at 68% [37].

In addition, this study reports more male cases than female, which supports Santinelli et al.’s [32] findings, but not Beccati et al.’s [17] or May-Davis et al.’s [36]. However, with such a small sample size (*n* = 20) this result is inconclusive. In addition, no breed association was found, contrary to the findings of May-Davis [46], where a strong relationship to Thoroughbred horses was reported. Even so, Zimmerman et al. [47] noted a strong correlation between C6 aCLV and Thoroughbred sires in the breeding lines of modern German Warmbloods. With that said, 16/20 horses in this study did express Thoroughbred heritage at some point in their genealogy, showing a stronger connection to the Thoroughbred backline in breeding programs than previously reported [48,49,50].

Nineteen horses in this study had been ridden (R27 excluded), with an average of 5 riding years noted; here, 15/19 required higher skill sets of their riders, for example, eventing, dressage, and jumping. In consideration of the average 5-year riding career, this could imply that horses with C6 aCLV4 might struggle with harder skill sets for a longer period, especially with reports by riders of poor performance or not reaching their expected potential [17,19,20,43]. Physically, this might be a viable hypothesis for neck pain attributed to a C6/C7 congenital malformation, including one study where a tracheal ridge deformation was reported, as seen here [17,19,20,39,43]. Furthermore, a height > 15 hands demonstrated a greater number of cases than that for 14–15 hands, with none < 14 hands. However, smaller horses with C6 aCLV have been reported [17]. When considering size, several hypotheses could be extrapolated: smaller breeds are less affected; adult riders are more prevalent; or the chosen discipline requires a taller horse, and in these horses their Thoroughbred lineage might be a factor.

In addition, age was of concern in this study. A previous gross morphological study reported the average age at euthanasia in C6 aCLV cases as11.3 years [36]; yet, here the average age was two years lower, at 9.25. Potentially, this implies that C6 aCLV4 cases elicit more clinical relevance when considering disciplines requiring higher skill sets, especially as there exists a strong association between C6 aCLV4, C7 transposition, and neck pain. If so, for those competitive riders expecting extended careers with equine athletes, the radiographic protocols established in this study might be useful in a prepurchase examination.

The morphology of C6 CLV has been relatively static throughout evolution since Hyracotherium some 55 million years ago [3]; however, C6 aCLV has only been anatomically reported in the last 200–250 years [29,30]. Arnold [11] noted the importance of the CLV in mammalian evolution (including equines), describing it as a major attachment site for muscles that helps enable stereotypical neck posture and movement. Rombach et al. [42] reported that the C6 CLV is a primary attachment site for the thoracal portion of the longus colli muscle, where its reinforced strong tendon likely supports the ventral vertebral curvature of the cervicothoracic region, while Denoix et al. [51] mentioned its involvement in stabilizing and flexing the caudal neck. Considering these statements, normal cranial and thoracal longus colli morphology are vital in cervical movement, support, stabilization, and flexion. In support of a previous study by May-Davis and Walker [40], our findings here also indicate that in the asymmetrical form of unilateral C6 aCLV4 and C7 transposition cases, asymmetric function is likely. In consideration of the longus colli muscle variations, and likely dysfunction, the mention of neck pain might be related to compromised neuromotor control, as seen in humans [52].

Replication of the C7 foramen transversarium has previously been reported in horses [5,6,15,30,37] and humans [53,54]. In the 13 cases identified here, the vertebral artery deviated by ascending directly cranial to the first sternal rib and entered the caudal aspect of the replicated C7 foramen transversarium instead of C6. Deviations in the vertebral artery are not confined to equines; such variations have also been reported in canines and humans [53,54,55]. Even though clinical significance has not been investigated in equines, concerns have been reported in humans, where the variable anatomy of the vertebral artery could lead to intraoperative complications that could be life-threatening [55]. Therefore, we recommend in those horses displaying a C6 aCLV4 with C7 transposition that replication of the foramen transversarium and vertebral artery be further investigated before any procedures are undertaken in the region.

The limitations of the study were noted regarding the accuracy of radiographic angles; the size and conformation of the horses, especially shoulder placement; and defining the caudal border of the C6 transverse process from the foramen transversarium in some cases. When these difficulties were encountered, extra radiographs were taken to verify the structures of interest before euthanasia. Even so, the clarity of C7 was not apparent in all the radiographs, and the foramen transversarium in C7 was only evident through dissection. Regarding which, with no formal guide on variations in the longus colli muscle, the vertebral artery, or other structures in the vicinity, this process was extremely time-consuming, but necessary to acquire the accurate results needed for this study. Furthermore, the difference in the two radiographic units and sedation protocols might have impacted the consistency of imaging. Of particular importance is recognizing the limited number of cases in this study and, therefore, it is only an indication of what might be anatomically present in a C6 aCLV4 horse.

## 5. Conclusions

In this study, a radiographic guideline was established for identifying C6 aCLV4. By utilizing a lateral 30° dorsal–ventral oblique radiographic view and determining the caudal border of the transverse process’s connection to the vertebral body, this point of reference identified the cranial aspect of the adjacent caudal lamina ventralis. From here, a C6 aCLV could be evaluated and graded according to the size of the absent CLV, where a grade 4 aplasia displays no visible evidence of a caudal lamina ventralis. Furthermore, the previously reported strong dependency between the C6 aCLV4 transposing to the ventral surface of C7 was also demonstrated here. In addition, variations in the attachments of the longus colli muscles identified asymmetric hypertrophy that was more obvious in a unilateral presentation. Replication of the foramen transversarium in C7 also demonstrated a deviation in the vertebral artery contrary to normal anatomy.

These variations imply the need for further investigation in live horses after a radiographically diagnosed C6 aCLV4, especially when neck pain is indicated. Additionally, these findings might help with isolating neck pain in the caudal neck while assisting in anatomical referencing for surgical procedures in the region. Therefore, in consideration of the morphological variations observed in this study, it is a recommendation of the authors to conduct further studies to map, isolate, and understand the potential functional ramifications associated with each variant.

## Figures and Tables

**Table 1:** Twenty horses with radiographs indicating a suspected C6 grade 4 aplasia of the caudal lamina ventralis met the criterion for the study. The description of each horse includes the breed, country, age, gender, discipline, years ridden, height range, and reason for euthanasia. ID represents identification—‘R’ for Rexos Incorporated and ‘A’ for Australia.

ID	Breed	Country	Age	Gender	Discipline	Years Ridden	Height Range (Hands High)	Reason for Euthanasia
			$\bar{x}$ = 9.25	13M/7F		$\bar{x}$ = 5		
R7	PRE	USA	14	M	Dressage	6	>15	RDB, PP, Deteriorating Health, Lameness
R14	QH	USA	10	M	HUS	8	>15	RDB, Shivers, PP
R15	WB	USA	6	F	Dressage	3	>15	RDB, Shivers, SD, PP Neurological
R20	TB	USA	7	F	OTTB/Ev	3	>15	RDB, SD, PP, Behavior
R21	WB	USA	8	F	Jumper	5	>15	RDB, IR, Neurologic, Behavior Hyperesthesia
R22	WB	USA	13	F	Dressage	5	>15	SD, Shivers, PP
R24	PRE	USA	5	M	Dressage	2	>15	Neurologic, PP
R26	WB	USA	19	F	Jumper	16	>15	Neurologic, Right Forelimb Lameness
R27	Fr/WB	USA	3	M	n/a	n/a	>15	Neurologic, Behavior, Hyperesthesia
R28	QH	USA	5	F	HUS	3	>15	PP, Right Forelimb Lameness
R29	Arab/Fr	USA	19	M	Dressage	15	>15	PP, Deteriorating Health
R30	Fr/WB	USA	6	F	Dressage	1	>15	Behavior
R35	WB	USA	22	M	Hunter	18	>15	Shifting Forelimb Lameness
R38	QH	USA	3	M	Hunter	1	>15	Shivers, SD, Neurological
R39	TB	USA	5	M	Eventing	2	>15	Forelimb Lameness, IR, Behavior
A1	ASH	AUS	10	M	PR	3	>15	RDB, Forelimb Neurological
A2	Brumby	AUS	8	M	PR	6	14–15	RDB, IRs
A3	ASH/TB/WMP	AUS	9	M	All Rounder	2	14–15	RDB, IR, Chronic Laminitis
A4	Conn/Tb	AUS	4	M	PR	1	>15	PP, Forelimb Neurological
A5	ASH	AUS	5	M	All Rounder	1	14–15	RDB, IR, Forelimb Neurological

Key—Breed: ASH (Australian Stock Horse), Conn (Connemara), Fr (Friesian), PRE (Pure Spanish Horse), QH (Quarter Horse), TB (Thoroughbred), WB (Warmblood), WMP (Welsh Mountain Pony); Key—Country: USA (United States of America), AUS (Australia); Key—Reason for Euthanasia: RDB (Random dangerous behavior), IR (Injured rider), PP (Poor performance), SD (Sleep depravation); Key—Gender: F (Female), M (Male); Key—Discipline: Ev (Eventing), HUS (Hunter under saddle), OTTB (Off-the-track thoroughbred), PR (Pleasure riding).

**Table 2:** Radiographs comparable to the gross morphology of a C6 grade 4 aplastic caudal lamina ventralis in twenty horses. The arrows located at the top right in the 2nd, 3rd, and 4th columns are directed cranially. The white asterisk in the 2nd column identifies the trachea on the ventral line of the caudal vertebrae. The red arrow in the 3rd and 4th columns indicate the caudal border of the transverse process, and anatomically caudal to this bony landmark, the C6 grade 4 aplasia of the caudal lamina ventralis is evident.

ID	C6 Radiograph	Caudal Border of the Transverse Process	C6 Gross Morphology Bone View	C6 aCVL
Normal C6—Left view Thoroughbred 14-year-old Gelding			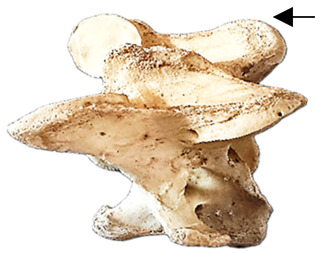	N/A
R7	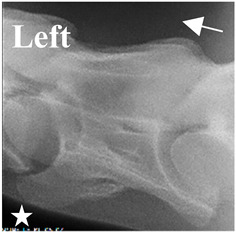	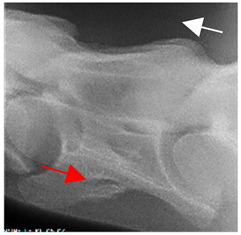	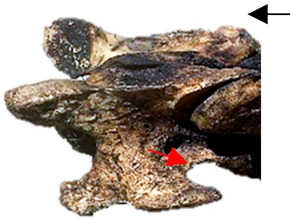	Left
R14	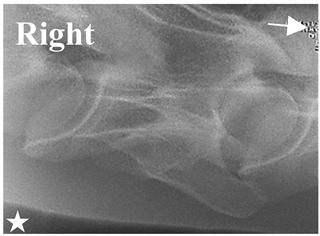	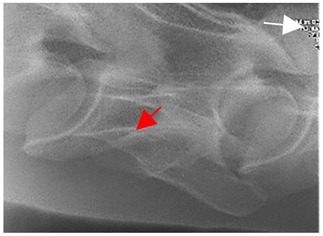	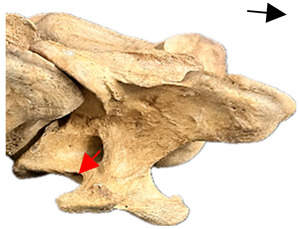	Bilateral
R15	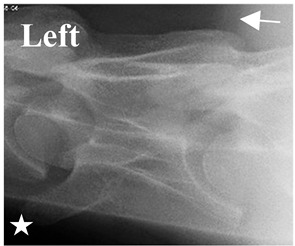	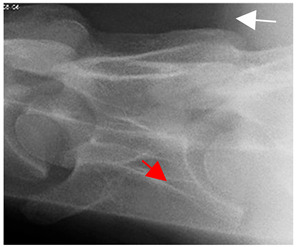	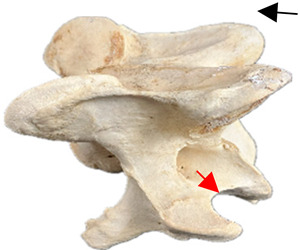	Bilateral
R20	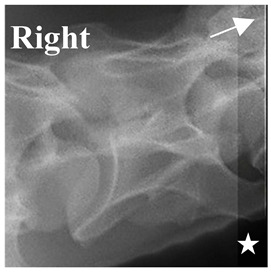	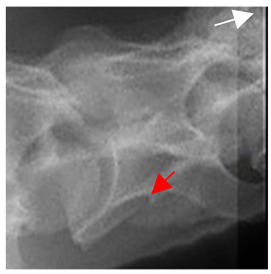	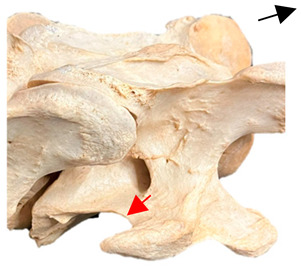	Bilateral
R21	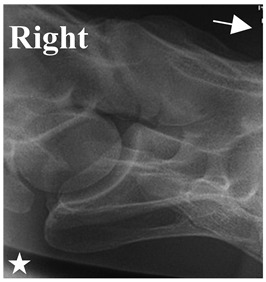	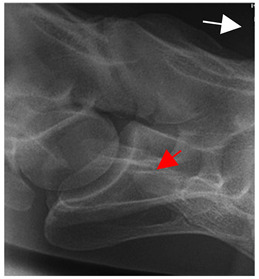	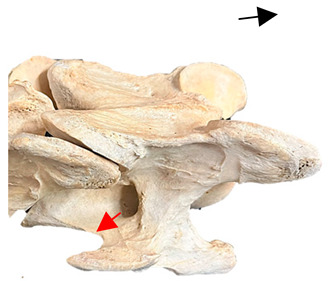	Right
R22	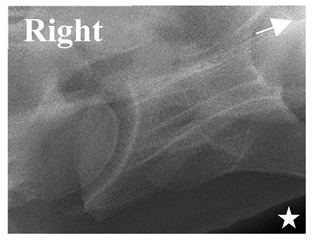	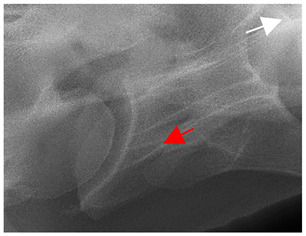	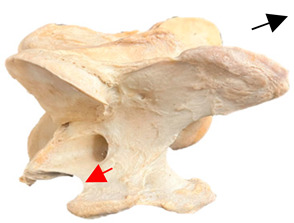	Bilateral
R24	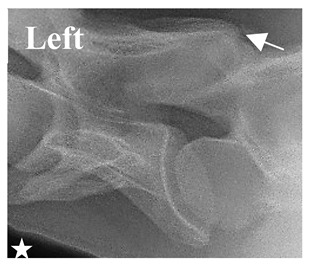	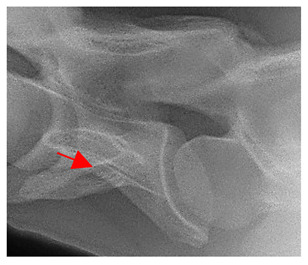	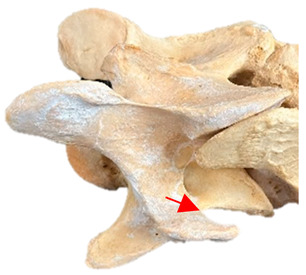	Bilateral
R26	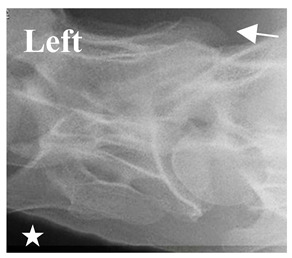	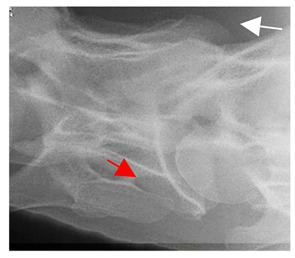	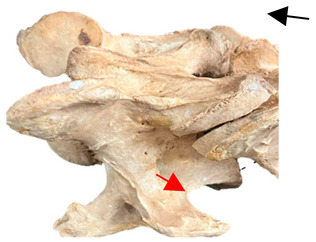	Bilateral
R27	** 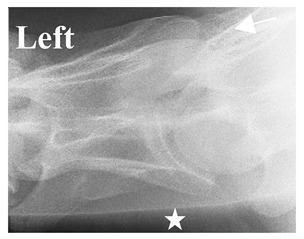 **	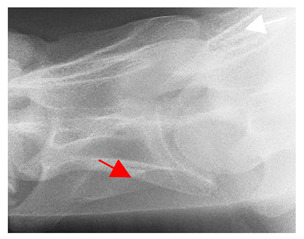	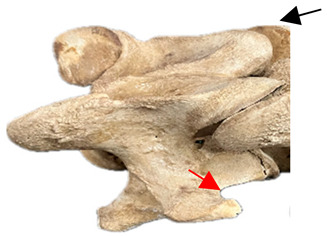	Bilateral
R28	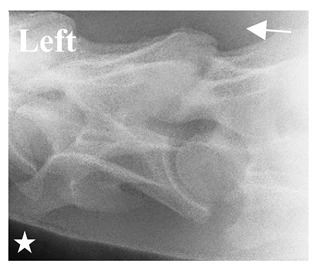	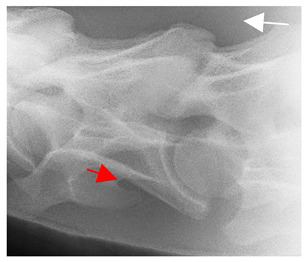	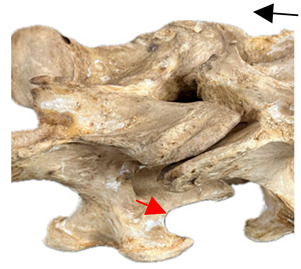	Bilateral
R29	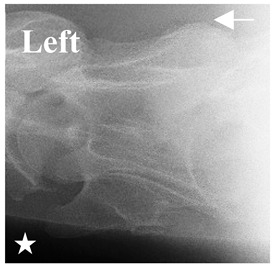	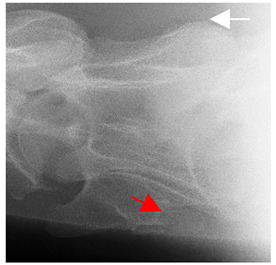	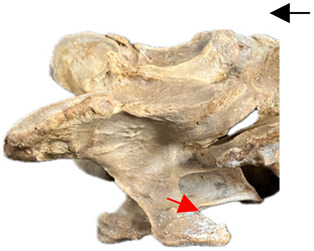	Left
R30	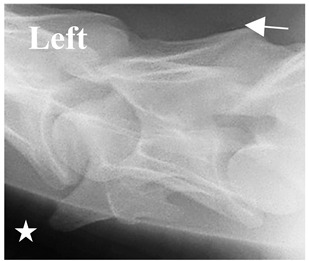	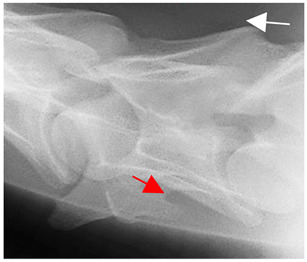	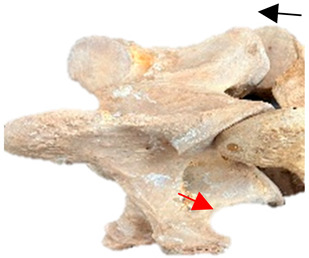	Left
R35	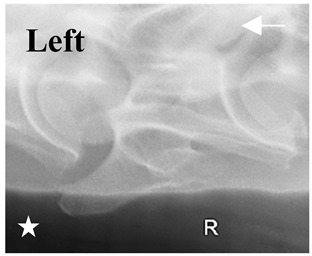	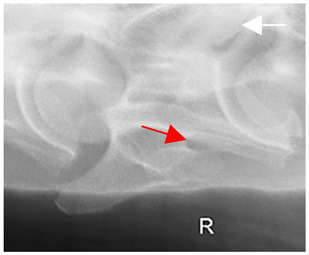	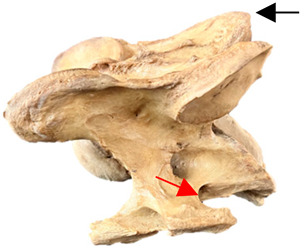	Left
R38	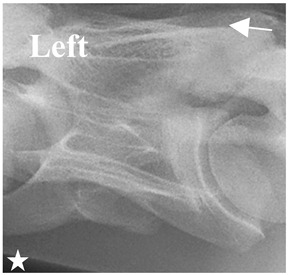	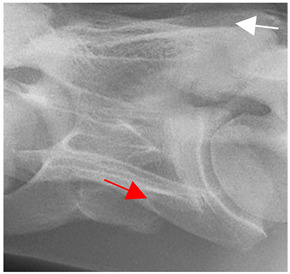	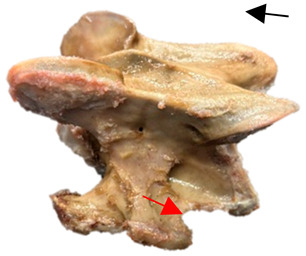	Bilateral
R39	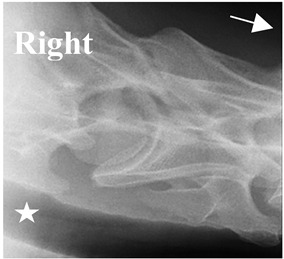	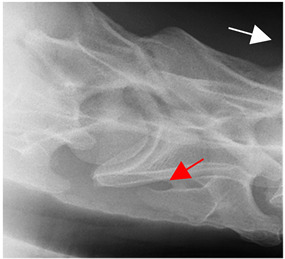	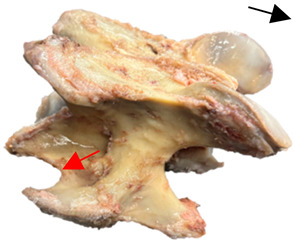	Bilateral
A1	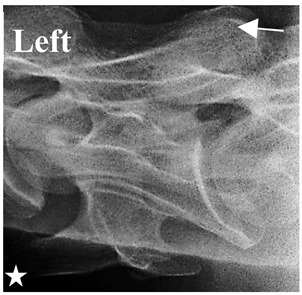	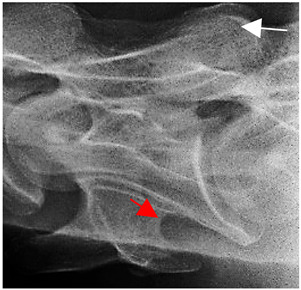	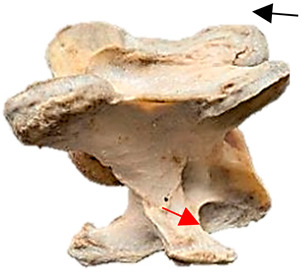	Left
A2	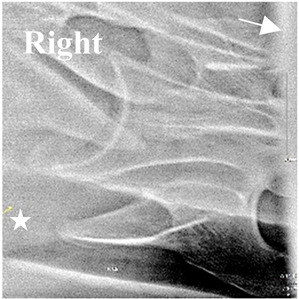	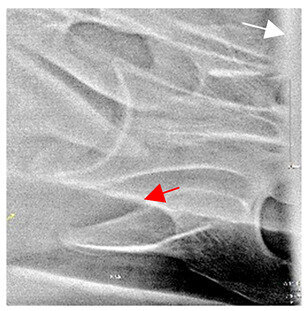	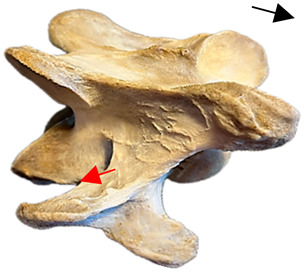	Right
A3	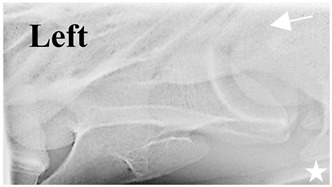	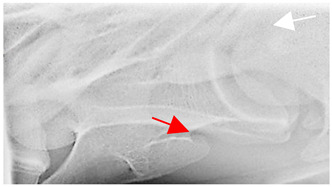	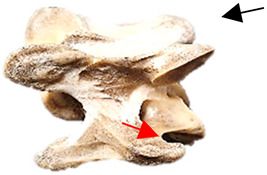	Bilateral
A4	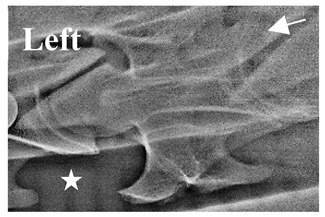	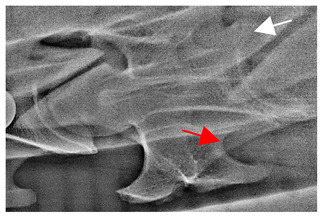	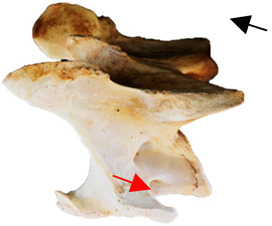	Left
A5	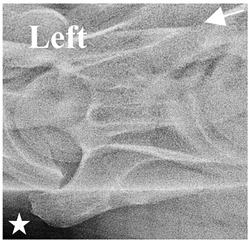	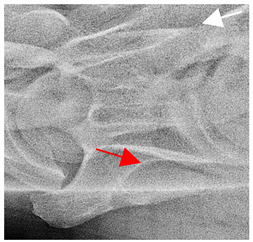	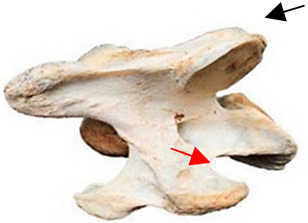	Bilateral

**Table 3:** Gross morphology findings of the C6 grade 4 aplastic caudal lamina ventralis cases, including transpositions to C7 and replication of the foramen transversarium.

ID	Breed ID	Age	Sex	Years Ridden	Height Range	C6 aCVL4	C7 Transposition	C7 Replication Foramen Transversarium Cranial Bone View
		$\bar{x}$ = 9.25	13M/7F	$\bar{x}$ = 5		B—12/20 U—8/20	19/20	13/20
Normal C7—Cranial view Thoroughbred 14-year-old Gelding								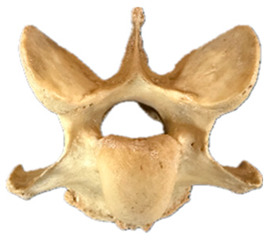
R7	PRE	14	M	6	>15	Left	Left	None
R14	QH	10	M	8	>15	Bilateral	Bilateral	None
R15	WB	6	F	3	>15	Bilateral	Bilateral	Bilateral 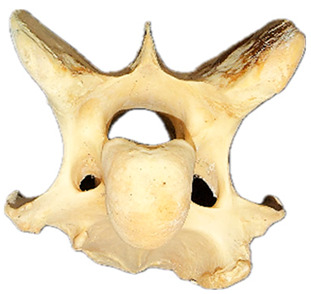
R20	TB	7	F	3	>15	Bilateral	Bilateral	Bilateral 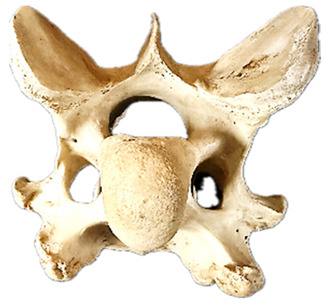
R21	WB	8	F	5	>15	Right	Right	Right 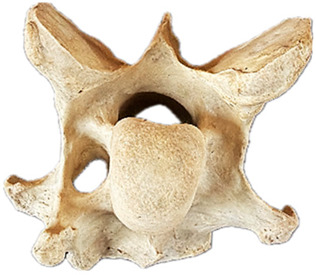
R22	WB	13	F	5	>15	Bilateral	Bilateral	Right 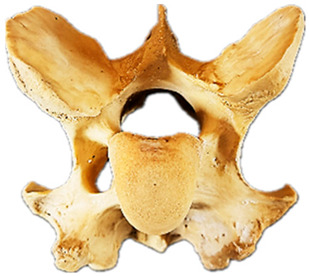
R24	PRE	5	M	2	>15	Bilateral	Bilateral	None
R26	WB	19	F	16	>15	Bilateral	Bilateral	Bilateral 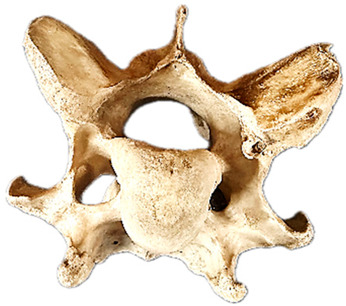
R27	Fr/WB	3	M	n/a	>15	Bilateral	Bilateral	Bilateral 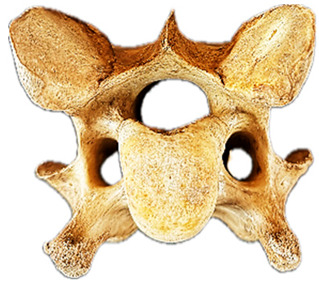
R28	QH	5	F	3	>15	Bilateral	Bilateral	Bilateral 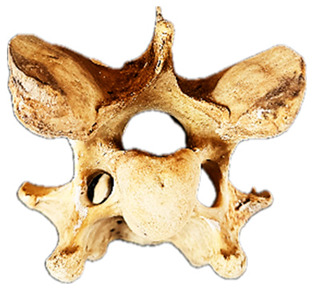
R29	Arab/Fr	19	M	15	>15	Left	Left	None
R30	Fr/WB	6	F	1	>15	Left	Left	Left 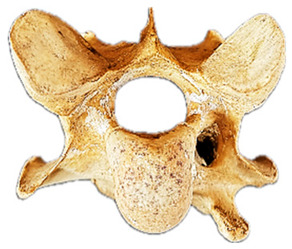
R35	WB	22	M	18	>15	Left	Left	Left 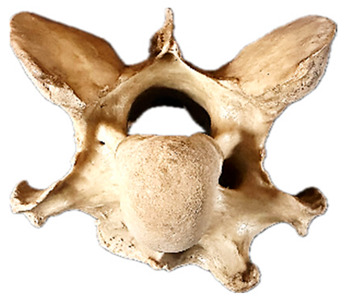
R38	QH	3	M	1	>15	Bilateral	Left	Left 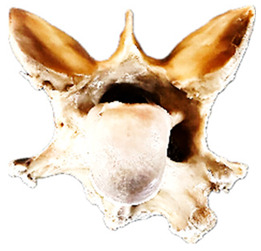
R39	TB	5	M	2	>15	Bilateral	Bilateral	Left 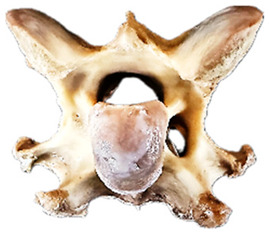
A1	ASH	10	M	3	>15	Left	None	None
A2	Brumby	8	M	6	14–15	Right	Right	None
A3	ASH/TB/WMP	9	M	5	14–15	Bilateral	Bilateral	Bilateral—N/A
A4	Conn Sport/TB	4	M	1	>15	Left	Left	None
A5	ASH	5	M	1	14–15	Bilateral	Left	Left—N/A

Key—Breed: ASH (Australian Stock Horse), Conn (Connemara), Fr (Friesian), PRE (Pure Spanish Horse), QH (Quarter Horse), TB (Thoroughbred), WB (Warmblood), WMP (Welsh Mountain Pony); Key—Country: USA (United States of America), AUS (Australia); Key—Gender: F (Female), M (Male).

## Data Availability

The original contributions presented in this study are included in the article. Further inquiries can be directed to the corresponding authors.
